# mTOR inhibitor everolimus reduces invasiveness of melanoma cells

**DOI:** 10.1007/s13577-019-00270-4

**Published:** 2019-10-04

**Authors:** Dorota Ciołczyk-Wierzbicka, Dorota Gil, Marta Zarzycka, Piotr Laidler

**Affiliations:** Chair of Medical Biochemistry, Jagiellonian University Medical College, ul. Kopernika 7, 31-034 Kraków, Poland

**Keywords:** Melanoma, Cell invasion, Protein kinase inhibitors, mTOR

## Abstract

The mammalian target of rapamycin (mTOR) plays a key role in several cellular processes: proliferation, survival, invasion, and angiogenesis, and therefore, controls cell behavior both in health and in disease. Dysregulation of the mTOR signaling is involved in some of the cancer hallmarks, and thus the mTOR pathway is an important target for the development of a new anticancer therapy. The object of this study is recognition of the possible role of mTOR kinase inhibitors—everolimus single and in combination with selected downstream protein kinases inhibitors: LY294002 (PI3 K), U0126 (ERK1/2), GDC-0879 (B-RAF), AS-703026 (MEK), MK-2206 (AKT), PLX-4032 (B-RRAF) in cell invasion in malignant melanoma. Treatment of melanoma cells with everolimus led to a significant decrease in the level of both phosphorylated: mTOR (Ser2448) and mTOR (Ser2481) as well as their downstream effectors. The use of protein kinase inhibitors produced a significant decrease in metalloproteinases (MMPs) activity, as well as diminished invasion, especially when used in combination. The best results in the inhibition of both MMPs and cell invasiveness were obtained for the combination of an mTOR inhibitor— everolimus with a B-RAF inhibitor—PLX-4032. Slightly less profound reduction of invasiveness was obtained for the combinations of an mTOR inhibitor—everolimus with ERK1/2 inhibitor—U126 or MEK inhibitor—AS-703026 and in the case of MMPs activity decrease for PI3 K inhibitor—LY294002 and AKT inhibitor—MK-2206. The simultaneous use of everolimus or another new generation rapalog with selected inhibitors of crucial signaling kinases seems to be a promising concept in cancer treatment.

**Electronic supplementary material:**

The online version of this article (10.1007/s13577-019-00270-4) contains supplementary material, which is available to authorized users.

## Introduction

Tumor cell migration and invasion that play fundamental roles in cancer metastasis are highly complicated, multi-stage processes with several signaling pathways and proteins involved in it. One of them includes PI3 K/AKT and high likely mTOR kinases [1].

**Fig. 1:**
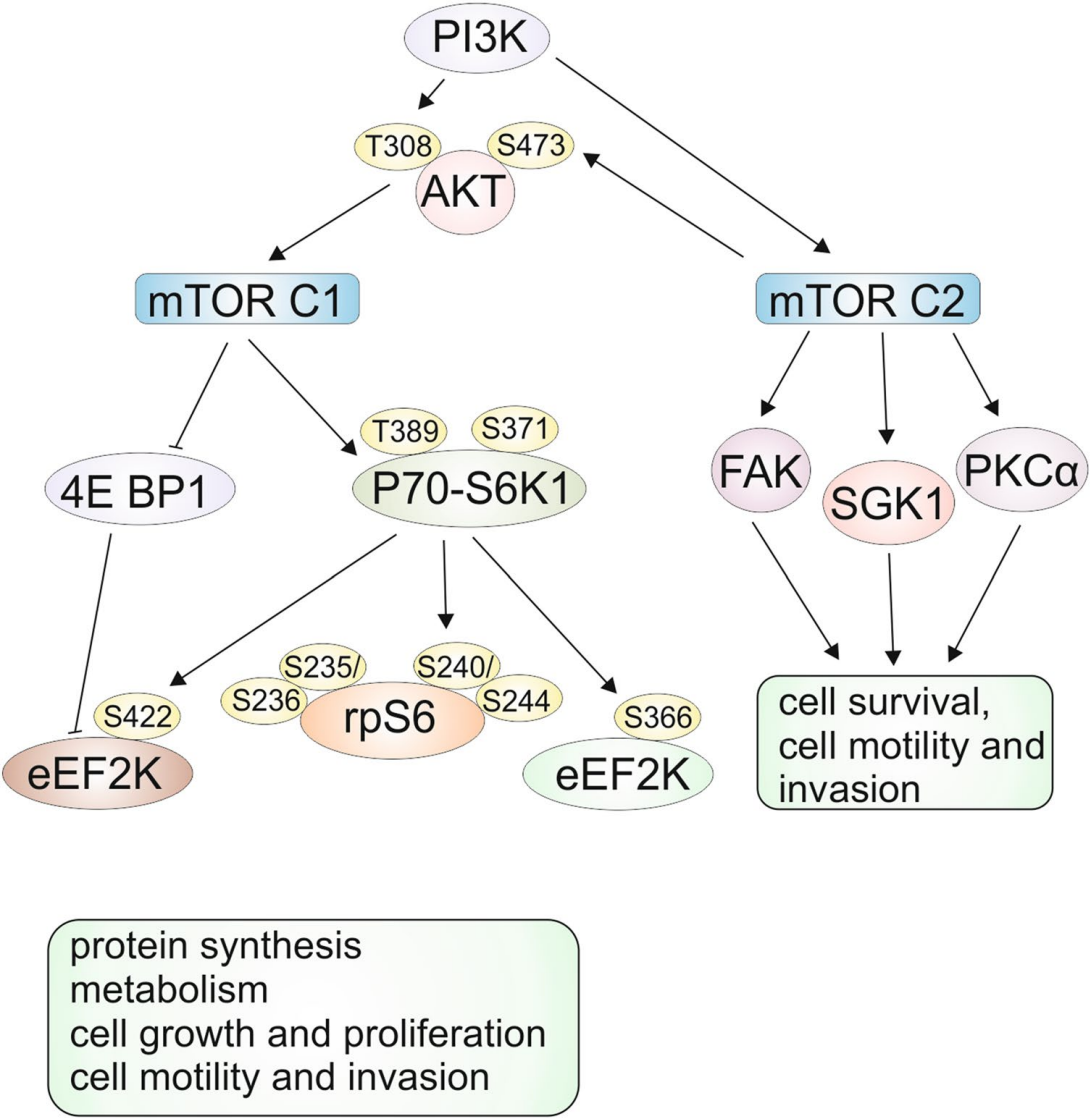
mTOR signaling pathways. mTOR (mammalian target of rapamycin) protein forms two unique complexes, called mTORC1 and mTORC2. mTORC1 regulates numerous processes by phosphorylation of p70-ribosomal protein S6 kinase 1 (p70-S6K1) and elongation initiation factor (EIF)-4E binding protein 1 (4E-BP1). Eukaryotic elongation factor 2 kinase (eEF2 K). mTORC2 controls cell structure, cytoskeletal reorganization, and survival by activating serum and glucocorticoid kinase (SGK1), focal adhesion kinase (FAK), protein kinase B (AKT), and protein kinase C α (PKCα) based on [1, 3, 5]

mTOR (the mammalian target of rapamycin) is a serine/threonine kinase that includes two distinct multi-component complexes, mTORC1 and mTORC2 [2], interacting with each other [3], and plays a central role in cell growth, proliferation, differentiation, motility, invasion, and survival [1, 2]. The overview of signaling pathways including mTORC1 and mTORC2 shown in Fig. 1, clearly indicates the phosphorylation of among other ribosomal protein S6 kinase (p70-S6K1) and elongation initiation factor (EIF)–4E binding protein 1 (4E-BP1) by mTORC1 complex. mTORC1 complex regulates cell growth, proliferation, migration, and invasion [1, 2]; moreover, overexpression of downstream mTORC1 effectors (p70-S6K1 and 4E-BP1) leads to poor cancer prognosis [2].

mTORC2 complex via protein kinase B (AKT) [2] participates in the regulation of such processes as cell survival and cytoskeletal organization by activating serum and glucocorticoid kinase (SGK1), focal adhesion kinase (FAK), and protein kinase C α (PKCα) [1].

In addition to its link to cancer, the mTOR pathway regulates major cellular processes and is implicated in several other pathological conditions such as obesity, type 2 diabetes, and neurodegeneration [4].

Since mTOR may be abnormally regulated in tumors signaling pathways, targeting either mTORC1 or mTORC2 has been spotlighted as one of the major anticancer strategies [2]. The effects of the combined use of rapalogs with other anticancer agents or rapalogs alone are under investigation in several human cancers, such as brain, breast, and other solid tumors [5].

The data of Conciatori et al. [3] as well as our previous studies on the use of protein kinase inhibitors in melanoma cells confirmed the efficacy of mTOR inhibitors: rapamycin and everolimus in inhibiting their proliferation and cell cycles [6], induction of apoptosis and in combination with knock down of N-cadherin gene decreased invasiveness [7]. Our last studies [8] have also demonstrated the efficacy of mTOR everolimus inhibitor in combination with MEK kinase inhibitor—AS-703026 or AKT kinase inhibitor—MK-2206 in the induction of apoptosis in melanoma cells. It seems, therefore, important to undertake research on the use of a combination of protein kinase inhibitors with particular emphasis on mTOR inhibitor everolimus in reducing the invasiveness of melanoma cells. The data presented herein points to the crucial role of mTOR signaling in cancer progression, as well as the prospect of implementation of a successful combination of its inhibitors in cancer treatment.

## Materials and methods

### Cell culture

Human melanoma cell lines: WM793 (VGP-vertical-growth phase); Lu1205 (metastatic; developed in mice in response to injection of primary VGP WM793 cells; biopsy taken from mouse lungs); WM115 (VGP-vertical growth phase) and WM266-4 (metastatic; derived from right thigh skin) were obtained from the ESTDAB Melanoma Cell Bank (Tubingen, Germany). These cell lines feature a BRAF V600E (WM793, Lu1205), V600D (WM115 and WM266-4) mutation, as well as hemizygous deleted PTEN and CDK4 K22Q mutation, wild type for N-ras, and c-KIT. Cells were cultured in RPMI-1640 medium supplemented with 10% fetal bovine serum and antibiotics: penicillin, streptomycin. Cells were incubated at 37 °C in a humidified atmosphere of 5% CO_2_ in the air. Cells were treated with inhibitors of: 1/PI3 K–LY294002 (Cell signalling TM) at 20 μM concentration, 2/ERK1/2–U0126 (Cell Signalling TM) at 10 μM concentration, 3/mTOR–everolimus (Selleck) at 5 nM concentration, 4/B–RAF-GDC-0879 (Selleck) at 2 μM concentration, 5/MEK–AS-703026 (Selleck) at 10 μM concentration, 6/AKT–MK-2206 (Selleck) at 2 μM concentration, and 7/B–RRAF-PLX-4032 (Vemurafenib) (Selleck) at 10 μM concentration. The incubation time of melanoma cells with inhibitors were 24 and 48 h.

### Cell invasion assay

Cell invasion assays were performed using conventional Boyden transwell methods in keeping with the manufacturer’s protocol (BD BioCoat™ FluoroBlok Invasion System No. 354166) described previously [7].

### Zymography

Preparation of samples for gelatin zymography and densitometry analyses of gelatinolytic activities of metalloproteinases: MMP-2 and MMP-9 was described previously [7].

### Western blot analysis

The Western blot method was previously described [9]. Antibodies against: mTOR (7C10) #2983, Phospho-mTOR (Ser2448) (D9C2) #5536, Phospho-mTOR (Ser2481) #2974, Phospho-p70 S6 kinase (Ser371) #9208, Phospho-p70 S6 kinase (Thr389) (108D2) #9234, p70 S6 kinase (49D7) #2708, phospho-S6 ribosomal protein (Ser235/236) (D57.2.2E) #4858, phospho-S6 ribosomal protein (Ser240/244) (D68F8) #5364, S6 ribosomal protein (5G10) #2217 (Cell Signaling Technology), N-cadherin #610920, Vimentin #550513, Phospho-FAK (pY397) #611722, FAK #610088 (BD Biosciences), and β-actin (A2228, SIGMA) were used to detect indicated proteins. Bands were visualized using horseradish peroxidase-coupled secondary anti-mouse or anti-rabbit antibody (Cell Signaling Technology). Immunoreactivity proteins were detected using chemiluminescence and images were captured with a ChemiDoc MP Imaging System (Bio-Rad Labs). To obtain quantitative results, immunoblots were scanned using SynGene Gene Tools version 4.03.0 (Synoptics Ltd Beacon House, Nuffield Road Cambridge, CB4 1TF, UK). Densitometry was used to normalize to the β-actin protein level. Presented are representative membranes of at least three independent experiments with similar results.

### Statistics

Cell invasion data were calculated from the mean values of repeated experiments. Statistical analyses were performed using one-way ANOVA with post hoc Tukey test (Statistica 12.0 StatSoft); indicates a significant difference: *p < 0.05, **p < 0.005, ***p < 0.00005.

## Results

### Everolimus regulates signaling pathway

We investigated the effect of everolimus (mTOR inhibitor) on the signaling pathway associated with the mTORC1 and mTORC2 complexes in melanoma Lu1205 and WM793 cell lines. mTORC1 is involved in the regulation of a range of cellular functions such as proliferation, survival, activation, differentiation, and migration, while mTORC2 controls cell structure, cytoskeletal reorganization, cell migration, and survival (Fig. 1).

**Fig. 2:**
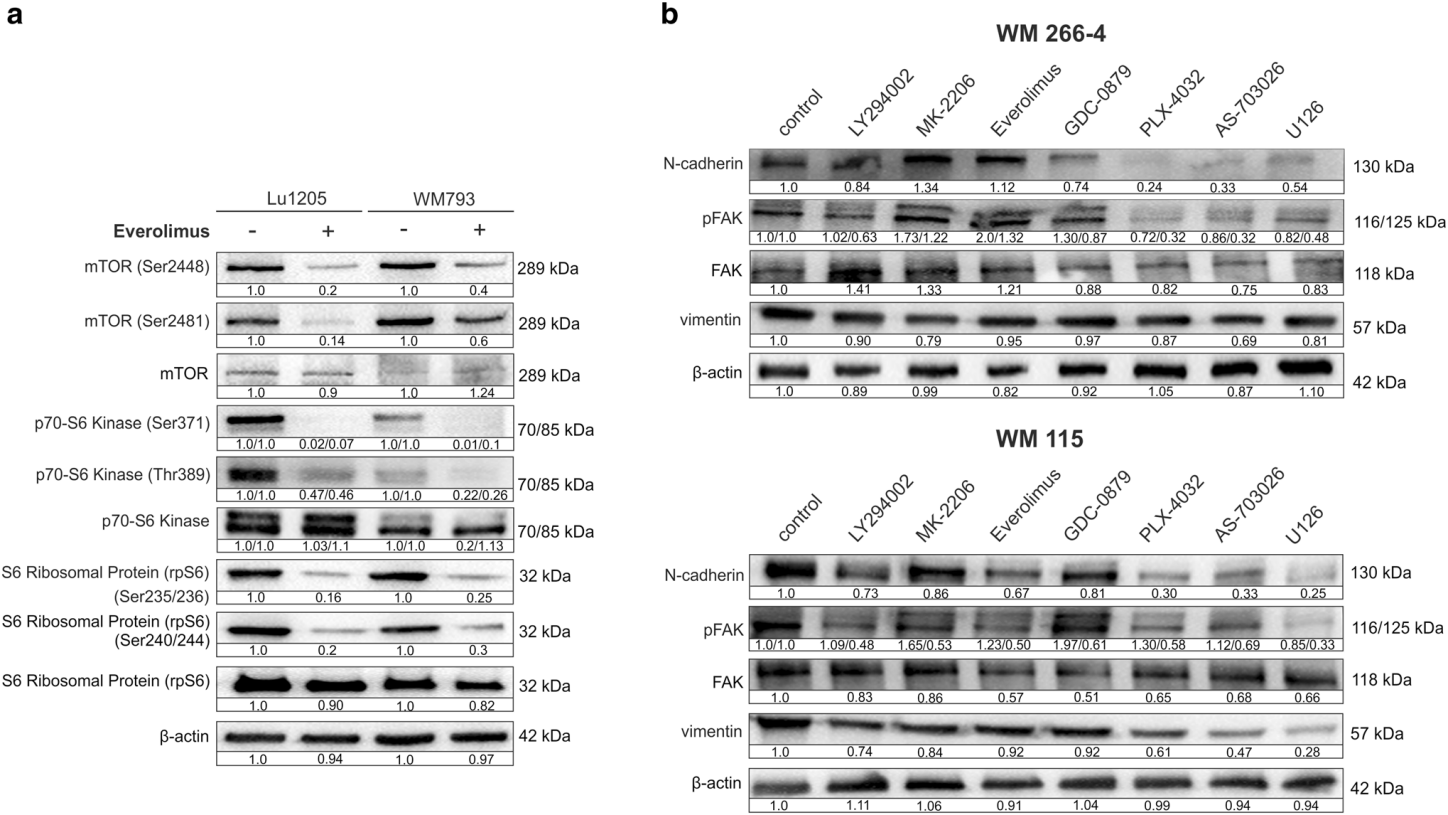
Western-blot of mTOR signaling protein expression in melanoma cells: Lu1205 and WM793 (**a**). The effect of protein kinase inhibitors on cell adhesion proteins in melanoma cells (**b**). Densitometry analyses of western blot were performed on raw volume (sum of intensities of bound-volume calculated from the area of the peak). Densitometry was used to normalize to the β-actin protein level and to control (melanoma cells untreated with protein kinase inhibitors). Presented are representative of at least three independent experiments with similar results

Western blot analysis of the phosphorylated mTOR: phospho-mTOR (Ser2448), phospho-mTOR (Ser2481), and total mTOR are presented in Fig. 2a. Treatment of melanoma cells with 5 nM everolimus resulted in the decrease of the level of phosphorylated mTOR protein (Ser2448)—associated with the complex mTORC1—by 80% in the case of the Lu1205 line, and by 60% in the WM793 line (Fig. 2a).

The use of everolimus also reduced the level of phosphorylated mTOR (Ser2481) associated mainly with a second mTOR complex (mTORC2) without affecting the expression of total mTOR protein (Fig. 2a).

We also studied the effect of everolimus on the expression of Ribosomal protein S6 kinase (S6K1, known as p70-S6K1) and S6 Ribosomal Protein (rpS6). We observed significant decrease (above 95%) in expression of both isoforms (70 kDa and 85 kDa) of p70 S6 kinase (Ser371) and over 50% decrease in the case of p70-S6 (Thr389) for both cell lines Lu1205 and WM793 without meaningful effect on the expression of the total level of p70-S6 kinase (Fig. 2a).

Expression of S6 ribosomal protein (Ser235/236) and S6 ribosomal protein (Ser240/244) decreased in the case of both melanoma cell lines by about 70–80% without meaningful effect on the expression of total level of S6 ribosomal protein (Fig. 2a).

We also tested the influence of protein kinase inhibitors on the level of: N-cadherin, vimentin, focal adhesion kinase (FAK) and phospho-FAK (pY397). The most pronounced effect was obtained for the WM266-4 and WM115 lines (Fig. 2b). The greatest expression decrease of all the tested proteins was observed for the inhibitors of the MAP-kinase pathway (Fig. 2b). The most affected was expression of N-cadherins, which decreased especially upon treatment with MEK kinase inhibitor—AS-703026 by 50%, slightly lower for the B-RAF kinase inhibitors—PLX-4032 and ERK1/2 kinase—U126 by c. 40% (Fig. 2b). B-RAF kinase inhibitor, on the other hand –GDC-0879 had by and large no effect on the expression of the tested proteins (Fig. 2b).

### The effect of protein kinase inhibitors on the cell invasion

All tested melanoma cell lines manifested the ability for cellular migration towards the chemoattractant.

We did not observe any effect of cytotoxicity of the applied protein kinase inhibitors, alone or in combinations at the time of 72 h, LDH activity in the culture medium in no case exceeded 3.2%.

**Fig. 3:**
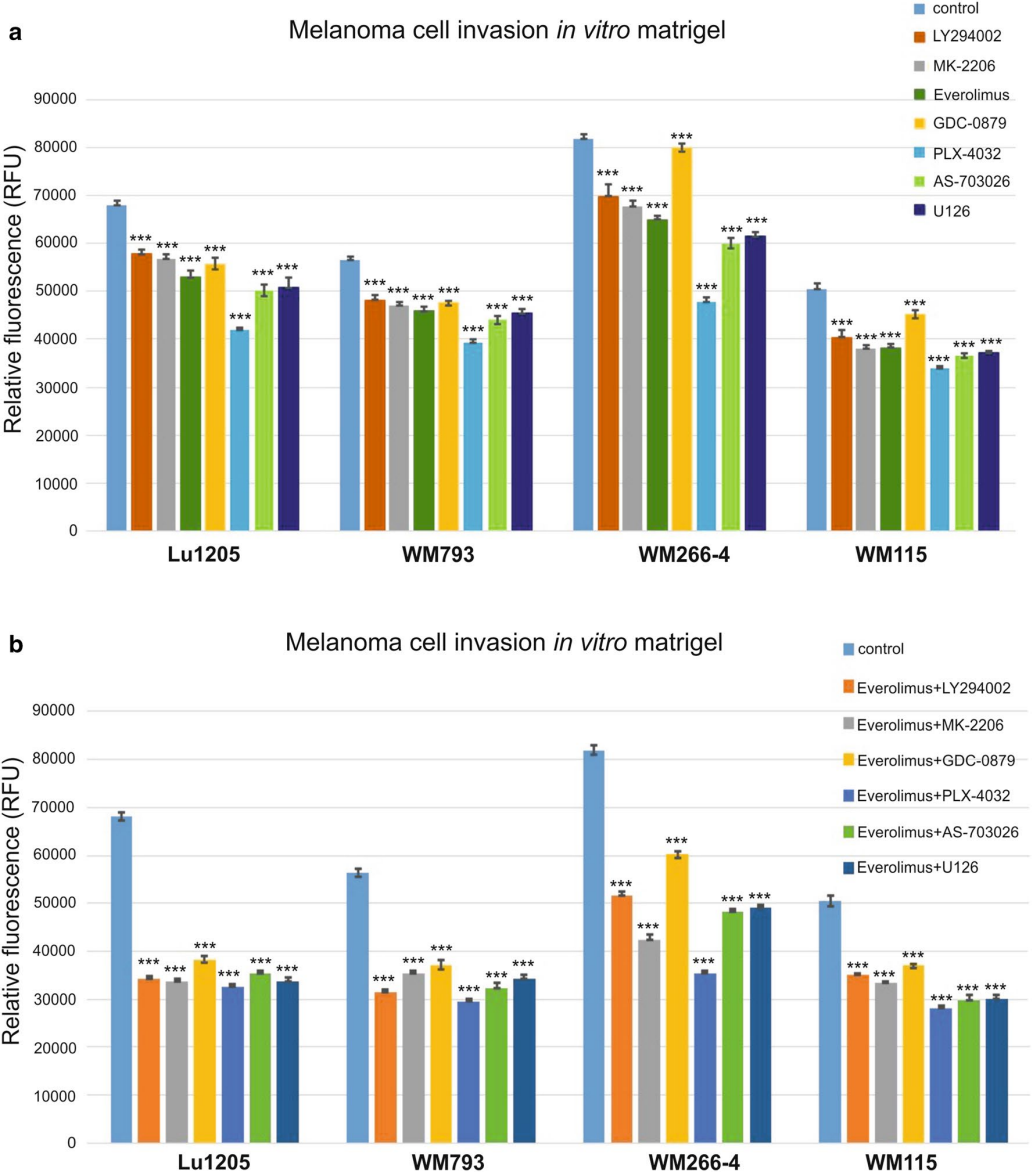
The effect of protein kinase inhibitors on melanoma cell invasion in vitro. Cell invasion assay through Matrigel-coated Boyden chamber. The histogram shows the quantification of cell invasion. Values are expressed as a mean ± standard deviation in 4 wells in two independent experiments. All results are presented as experimental mean values which were compared using one-way ANOVA with the Tukey’s post hoc test (Statistica ver. 12, StatSoft,); indicates a significant difference: *p < 0.05, **p < 0.005, ***p < 0.00005

Treatment of melanoma cells with single protein kinase inhibitors resulted in the decrease in cell invasion in vitro in the range of 10–41% relative to control cells in tested melanoma cell lines: Lu1205, WM793, WM266-4, and WM115 (Fig. 3).

WM115, WM266-4, and Lu1205 cells showed a high decrease of over 26% observed after the use of MEK kinase inhibitors: AS-703026 or U126 that was similar to the WM793 cell line response (Fig. 3). An analogous effect was observed after the use of the everolimus—mTOR inhibitor, which decreased invasion by approximately 23–24% (*p* < 0.00005) for the Lu1205 and WM115 cell lines, and 20% (*p* < 0.00005) for the WM266-4 and WM793 cell lines (Fig. 3).

The most significant decreases in cell invasion in vitro using a single inhibitor were observed in metastatic cell lines after application of an inhibitor of B-RAF kinase—PLX-4032. These decreases were, respectively 41% (*p* < 0.00005)—WM266-4, 38% (*p* < 0.00005)—Lu1205; slightly lower response for primary cell lines: 30–33% (*p* < 0.00005) for the WM793 and WM115 cells lines, respectively (Fig. 3). PI3 K/AKT inhibitors (LY294002, MK-2206) caused a reduction in the level of MMP-2 in the range of 15–17% in all of the tested cell lines (Fig. 3).

Treatment of melanoma cells with a combination of everolimus with other kinase protein inhibitors gave significantly better results than the use of single inhibitor for both cell lines. The reduction of invasiveness in vitro was in the range of 40–57% (Fig. 3).

Applications of everolimus inhibitor with an inhibitor: AS-703026, U126, LY294002, or MK-2206 has shown a decrease in in vitro invasiveness for each combination by approximately 50% for the Lu1205 and 40–45% for other cell lines. The combination of everolimus with the B-RAF kinase inhibitor—PLX-4032 resulted in the highest observed decrease in in vitro invasiveness which was in metastatic cell lines: 57% (*p* < 0.00005) for the WM266-4, 53% (*p* < 0.00005) for the Lu1205, and slightly lower for cells from primary phase: 48% (*p* < 0.00005) for the WM793, and 44% (*p* < 0.00005) for WM115 (Fig. 3).

### The effect of protein kinase inhibitors on gelatinolytic activities of the matrix metalloproteinases (MMPs): MMP-2 and MMP-9

**Fig. 4:**
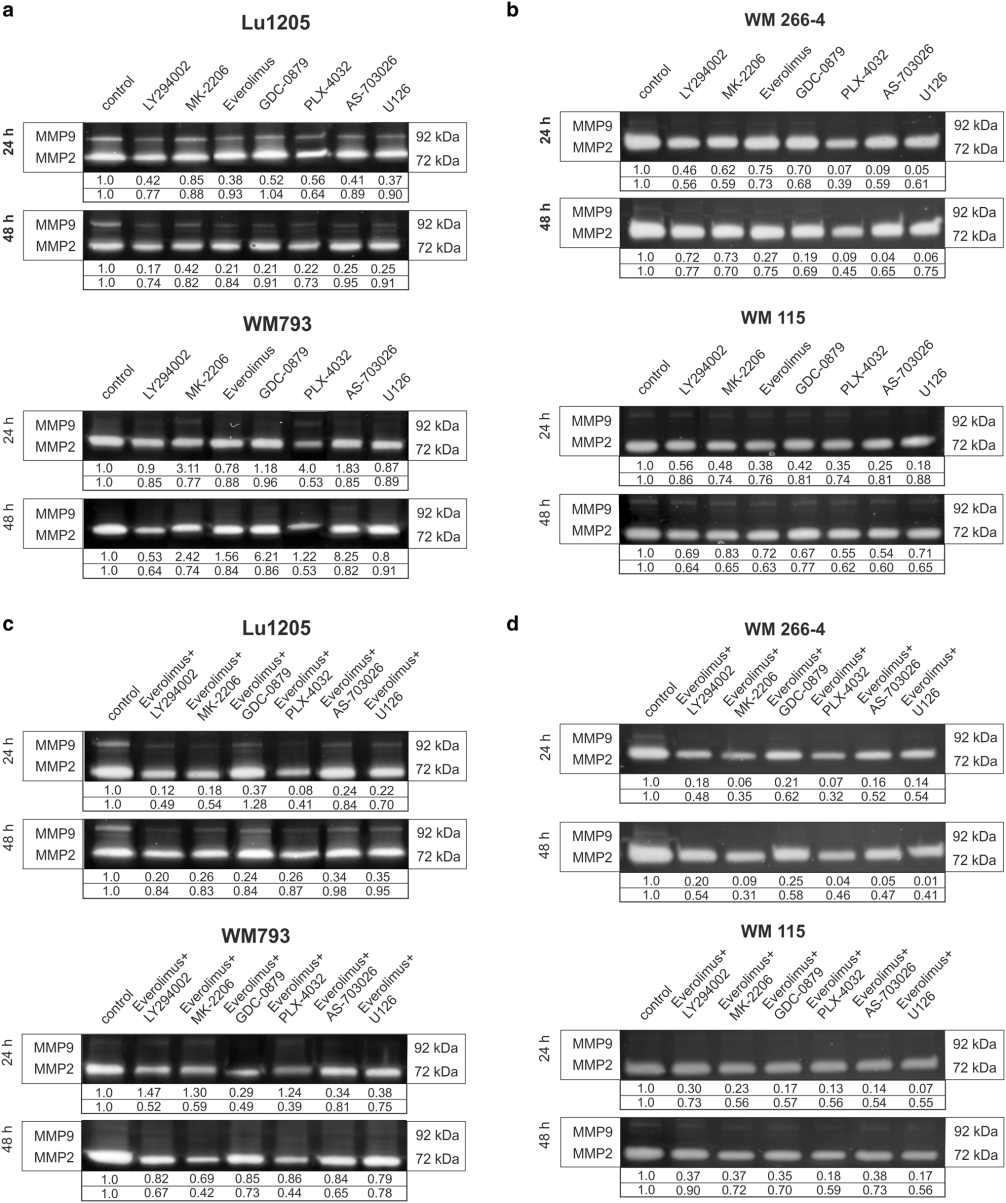
The effect of protein kinase inhibitors on gelatinolytic activities of MMP-2 and MMP-9 in melanoma cells. Densitometric analyses of MMP-2 and MMP-9 activities were performed on raw volume (sum of intensities of bound-volume calculated from the area of the peak). Presented are representative of at least three independent experiments with similar results

Activities of two metalloproteinases: MMP-2 and MMP-9 were studied in the primary (WM793, WM115) and metastatic (Lu1205, WM266-4) melanoma cell lines after 24-h and 48-h treatment with protein kinase inhibitors and their combinations (Fig. 4).

High level of MMP-2 metalloproteinase accompanied by lack of MMP-9 activity was found in the WM793, WM115, and WM266-4 cell lines, whereas the Lu1205 line manifested high activity of both MMP-2 and MMP-9 (Fig. 4, control lane).

The treatment of Lu1205 cells for 24 h with single inhibitors led to the highest decrease in the activity of MMP-9 in the case of U126 (ERK1/2 inhibitor)—63%, everolimus (mTOR inhibitor)—62% and AS-703026 (MEK inhibitor)—59% (Fig. 4a). After 48-h treatment with inhibitor/inhibitors, the highest decrease in MMP-9 activity was observed for LY294002 (PI3 K inhibitor), which reduced its activity by about 83% and for everolimus as well as GDC0879 and PLX-4032 (BRAF inhibitors)—80% each (Fig. 4a). A similar effect was observed for other melanoma cell lines (Fig. 4a, b), although it is difficult to interpret due to the very low expression of MMP-9.

The highest decrease in MMP-2 was observed for the WM266-4 and WM793 cell lines for which the expression of this metalloproteinase in the control lines was higher (Fig. 4a, b). The inhibitor most effective in reducing the activity of MMP-2, regardless of the time tested, was B-RAF inhibitor PLX-4032 kinase. It reduced the activity of MMP-2 in the studied melanoma lines by 60% for WM266-4, 50% for Lu1205, and at least about 35% for the WM115 line (Fig. 4a, b). Using the combination of protein kinases inhibitors with everolimus gave better results than the use of single inhibitors. For all tested cell lines, the best results in the case of MMP-2, as for MMP-9 were obtained by combining the mTOR inhibitor—everolimus with B-RAF inhibitor—PLX4032, which led to 60% decrease of MMP- 2 activity in all tested cell lines, and about 90% for the MMP-9 for the Lu1205 cell line (Fig. 4c, d).

Comparable decreases in MMP-9 and MMP-2 activity have been observed for the use of the combination of everolimus with an AKT inhibitor–MK-2206 or PI3 K—LY294002. For the WM266-4 and WM115 lines, the use of everolimus combinations with inhibitors of the kinase MAP pathway was also effective (Fig. 4c, d).

## Discussion

### mTOR signaling pathways

mTOR (the mammalian target of rapamycin)—plays a key role in homeostasis, integrates both intracellular and extracellular signals and serves as a central regulator of cell metabolism, growth, proliferation, invasion, and survival [3, 4, 10].

It has been previously demonstrated [6] that treatment of melanoma cells with mTOR inhibitors: rapamycin or everolimus had a significant effect on cell cycle regulation, and in consequence, proliferation [6, 8] as their use led to a significant reduction of the number of cancer cells. As many current studies focused—in the search for effective anticancer treatment—on new capabilities of already registered drugs and their use in combination with other potential anticancer ones, we decided to examine the effect of protein kinase inhibitors—in particular the very promising mTOR inhibitor—everolimus on cell invasion and metalloproteinase activity.

Treatment of melanoma cells: Lu1205 and WM793 with 5 nM concentration of everolimus resulted in a significant decrease in the level of phosphorylated mTOR (Ser2448) kinase associated with the activity of the mTORC1 complex. That result remains in accordance with the results of a large group of researchers confirming the effect of rapamycin and its analogs on the inhibition of mTORC1 complex activity [3, 11].

The use of the everolimus inhibitor also caused a decrease in the phosphorylated mTOR (Ser2481) level; a greater effect was observed for the Lu1205 line. The phosphorylated form of mTOR (Ser2481) is mainly associated with mTORC2, but it is also found in the mTORC1 complex [5]. Some literature data suggest that mTORC2 complex is insensitive to the rapamycin [12], while other studies [5, 13–15] report that rapamycin analogs (rapalogs) have an inhibitory effect on the mTORC2 complex, especially after prolonged treatment. Research based on rapalogs, among others with everolimus, have shown that observation of phosphorylation of mTOR (Ser2481) allowed for the correct prediction of the therapeutic efficacy of mTORC1 inhibitors against hepatocellular carcinoma [15].

We also tested the effect of everolimus on the expression of ribosomal protein S6 kinase (p70-S6K1) and S6 ribosomal protein (rpS6). We found a very large decrease (above 95%) in p70-S6 kinase (Ser371) and p70-S6 (Thr389) for both cell lines Lu1205 and WM793 without significant change in the total expression of p70-S6 kinase. Ribosomal protein S6 kinase, a critical mediator of cell growth, is overexpressed in many types of cancer and is related with their poor prognosis [9, 16]. Phosphorylation of S6 ribosomal protein (Ser235/236) and S6 ribosomal protein (Ser240/244) also very significantly decreased after treatment of melanoma cell lines with everolimus. According to the data of Wang and Fan [17], the phosphorylated form of p70-S6K1 (Thr389) represents the principal site of rapamycin-mediated inactivation of p70-S6K1 kinase and correlates closely with the loss of its activity.

According to Chen et al. [16], hyperphosphorylation of ribosomal protein S6 (rpS6) probably regulated by the AKT2/mTOR/p70-S6K1 pathway, signals unfavorable clinical survival in non-small cell lung cancer, especially in the early staged cases [16]. Chen et al. [16] also observed in human bronchial epithelial cell lines a relationship between induction of ribosomal protein S6 (rpS6) and a significant increase of cell migration, accompanied by enhancement of expression of P-paxillin, N-cadherin, vimentin, MMP-2, and reduction expression of E-cadherin, proteins which regulate cells adhesion and extracellular matrix degradation.

We also tested the influence of protein kinase inhibitors on the level of: N-cadherin, vimentin and focal adhesion kinase—FAK and p-FAK(Y397). In previous research we demonstrated that none of the tested cell lines showed E-cadherin expression, and we extensively researched the aspect of the influence of N-cadherin silencing on the process of proliferation, invasion and cell signaling in melanoma cells [7, 8]. Protein kinase inhibitors caused a decrease in the level of cell adhesion molecule, with the greatest effect observed for the inhibitors of B-RAF/MAP-kinase pathway.

### Protein kinase inhibitors and melanoma cell invasion

Treatment of the studied melanoma cell lines with protein kinases inhibitors, regarding the inhibition of cell invasiveness in vitro as well as the activity of metalloproteinases 2 and 9 gave the best results in the case of the use of B-RAF kinase inhibitor—PLX-4032. The primary cell line—WM793 and metastatic one—Lu1205, as in the majority of melanoma cases, carry a mutation in the B-RAF kinase (V600E), while the WM115 (primary) and WM266-4 (metastatic) cell lines have the V600D mutation, and all tested melanoma cell lines seem to be very sensitive to treatment with B-RAF inhibitors PLX-4032. Prominently weaker response, especially for the lines WM266-4 and WM115, was elicited by B-RAF—GDC-0879 inhibitor.

Slightly worse results were obtained while using ERK1/2 inhibitor—U126 and MEK inhibitor—AS-703026. In reducing the activity of metalloproteinase 2 and 9, more effective inhibitors were PI3 K—LY294002 and AKT—MK-2206. The use of everolimus gave a very significant effect in both cases.

Our former results [7] also confirm the satisfactory effect of inhibition of the invasiveness of melanoma cells using everolimus, in particular in combination with siRNA knockdown of the N-cadherin gene [7].

Research presented by Du et al. [18] suggests that everolimus inhibited growth, induced apoptosis, and arrested cell cycle of breast cancer cells via downregulation of PI3 K/AKT/mTOR signaling pathways. Our studies [8] also confirm that low nanomolar concentrations of the everolimus mTOR inhibitor in combination with the MEK kinase inhibitor AS-703026 or AKT kinase inhibitor-MK-2206 induce apoptosis in melanoma cells. They also found that everolimus showed great clinical efficacy in combination with tamoxifen in estrogen-receptor-positive breast cancer by inhibition of PI3 K and mTOR, which may further improve therapy via reduction of compensatory AKT activation [18]. The effects of the combined use of everolimus with other anticancer agents or rapalogs alone are under investigation in several human cancers, such as renal cell carcinoma, neuroendocrine carcinoma, breast cancer, glioblastoma multiform, and endometrial cancer, and have shown promising progression-free and overall survival in clinical studies [5]. Currently, temsirolimus and everolimus (mTOR inhibitors) are approved for use in Europe and the United States in a variety of cancer-related indications and are routinely used in oncology practices as an alternative to traditional cytotoxic chemotherapy [19]. The results obtained in the Phase II study clinical trials based on a group of 53 patients with unresectable metastatic melanoma [20] did not confirm the efficacy of everolimus (RAD-001) as a single-agent in the treatment, whereas in combination with temozolomide it showed promising clinical activity.

The benefit of everolimus-based therapy depends on the genetic status of mutations. According to Webber et al. [21] who based on research on molecular aberration predictive for response to everolimus, the use of mTOR inhibitor, regardless of tumor type, shows that the loss of function, aberration in Phosphatase and tensin homolog (PTEN) is associated with the success of therapy, while B-RAF wildtype could be responsible for the resistance. Preclinical data suggest that PTEN status may potentially be developed as one such biomarker of clinical situations in which combined inhibition of the MEK/ERK and PI3 K/AKT/mTOR pathways could be highly synergistic and require reduced single-agent doses of each agent, thereby reducing toxicity [3]. Examined herein melanoma cell lines: Lu1205, WM793 possessed B-RAF V600E, WM266-4 and WM115 V600D mutations as well as hemizygous PTEN deletion.

The highest reductions in the activity of metalloproteinases and the invasiveness of melanoma cells were obtained with the combination of the B-RAF inhibitor—PLX-4032 and mTOR inhibitor—everolimus. Similar observations were reported for A375M6 melanoma cell line [22].

Very promising results were also obtained for the use of a combination of the everolimus with the AKT inhibitors—MK-2206 and PI3 K kinase inhibitor—LY294002 (Fig. 4). Data presented by Yang et al. [23] confirmed the efficacy of simultaneous use of PI3 K/AKT/mTOR signaling pathway inhibitors in reducing cell proliferation, survival, and invasion in human colon cancer. The results of meta-analysis [24] which included 46 randomized controlled trials with a total of 15,511 patients and more than 100 arms suggest that the addition of PI3 K/AKT/mTOR pathway inhibitor to the therapy regimens for advanced solid tumors significantly improved the progression-free survival, especially among patients with breast cancer and neuroendocrine tumors, as well as those with PI3 K mutations. Several dual mTOR/PI3 K—kinase inhibitors, such as NVP-BEZ235, BEZ235, and GDC-0980, PF-04691502, XL765, GSK2126458, PI-103, and a different class of mTOR inhibitors (e.g., Torin1, PP242, PP30, Ku-0063794, WAY-600, WYE-687, WYE-354, and CC-223) are currently being developed for clinical use on the assumption that blockade of two different crucial nodes along the PI3 K signaling pathway might result in more complete pathway inhibition, disruption of pathway-reactivating feedback loops, and eventually enhanced anti-tumor activity [3].

Preclinical studies have also demonstrated that mTOR inhibitors—rapalogs may be related to the induction of signaling feedback loops limiting their anti-tumor effects [3, 25]. Simultaneous blocking of PI3 K, AKT, and mTOR is an effective method of tumor suppression by promoting prolonged AKT, S6K1, and 4E-BP1 dephosphorylation, and induction of apoptosis, which may reduce symptoms as well as improve patient response [3, 26].

The results presented herein seem to unanimously confirm that the idea of simultaneous use of rapalogs, especially everolimus and selected signaling kinase inhibitors, following recognition of the genetic status of cancer may bring a breakthrough in its treatment, and should soon be expected in clinical use.

## Electronic supplementary material

Below is the link to the electronic supplementary material.

Supplementary material 1 (TIFF 907 kb)

Supplementary material 2 (TIFF 923 kb)

Supplementary material 3 (TIFF 971 kb)

Supplementary material 4 (TIFF 982 kb)

Supplementary material 5 (DOCX 12 kb)

Supplementary material 6 (DOCX 12 kb)
