# Supplementary figures and images for: mTOR inhibitor everolimus reduces invasiveness of melanoma cells
Source: Hum Cell. 2019 Oct 4;33(1):88–97. doi: 10.1007/s13577-019-00270-4 (PMC6965047; doi:10.1007/s13577-019-00270-4)

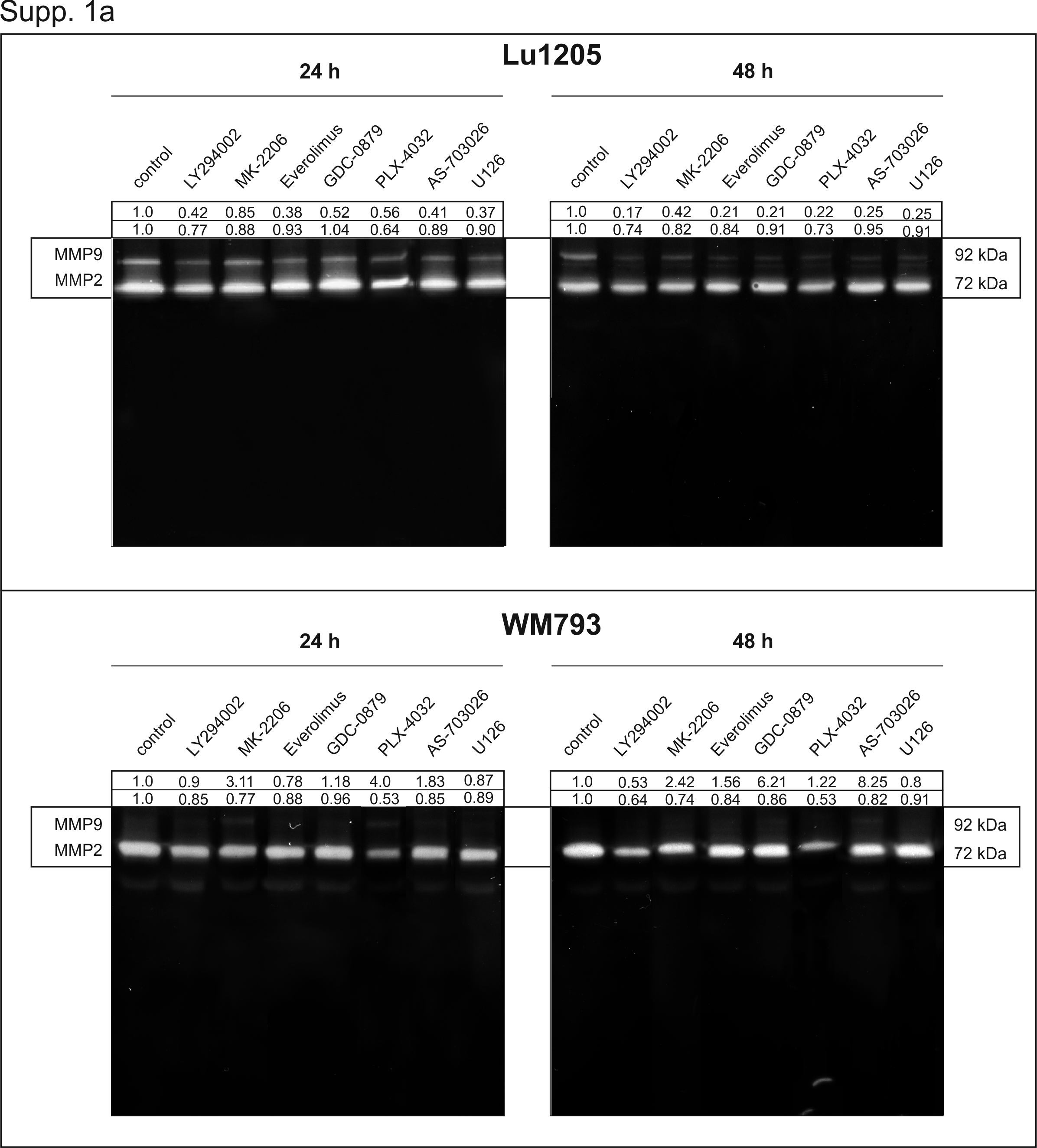

Supplement: Supplementary file 1 — Supplementary material 1 (TIFF 907 kb) [file 13577_2019_270_MOESM1_ESM.tif]

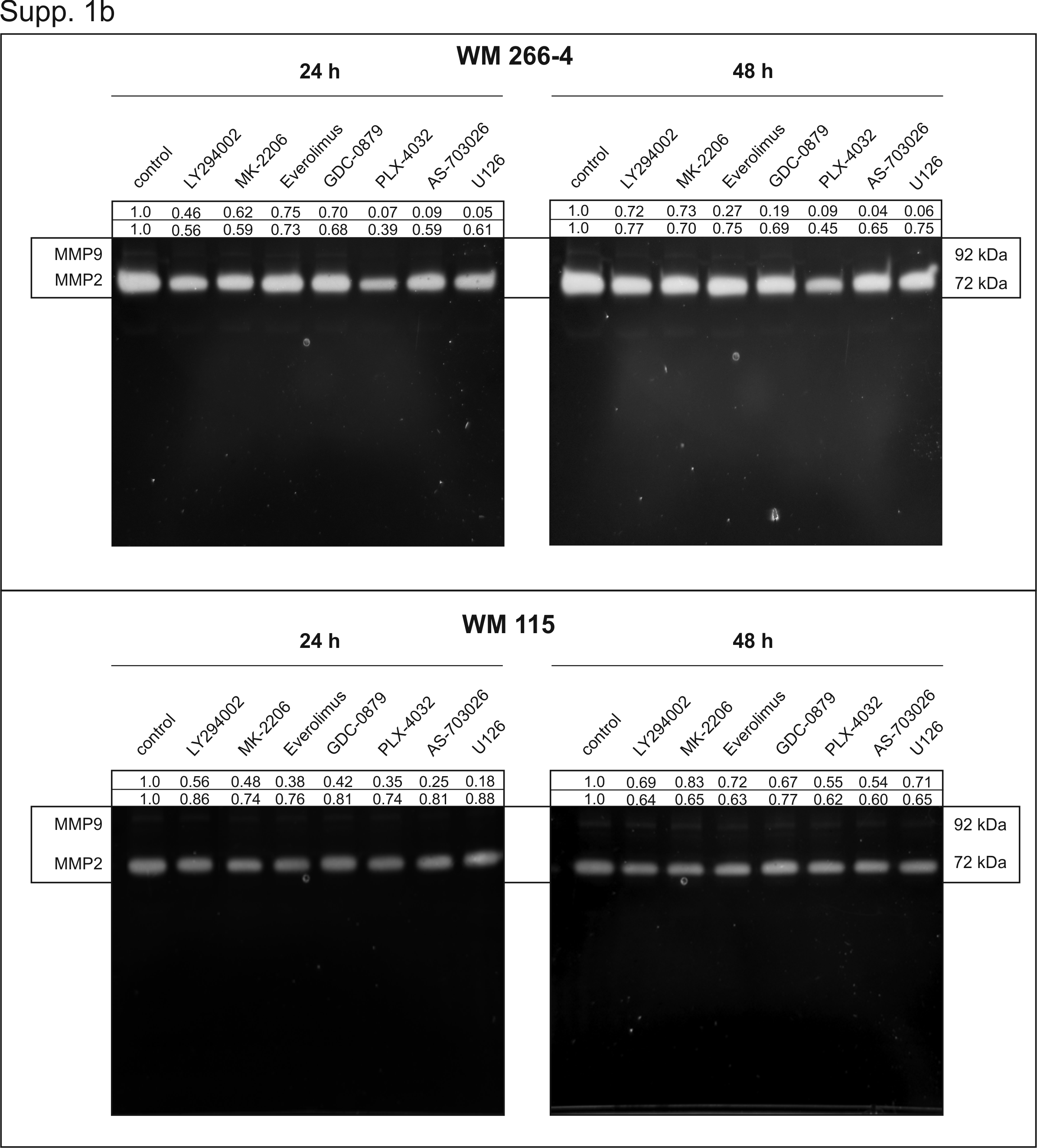

Supplement: Supplementary file 2 — Supplementary material 2 (TIFF 923 kb) [file 13577_2019_270_MOESM2_ESM.tif]

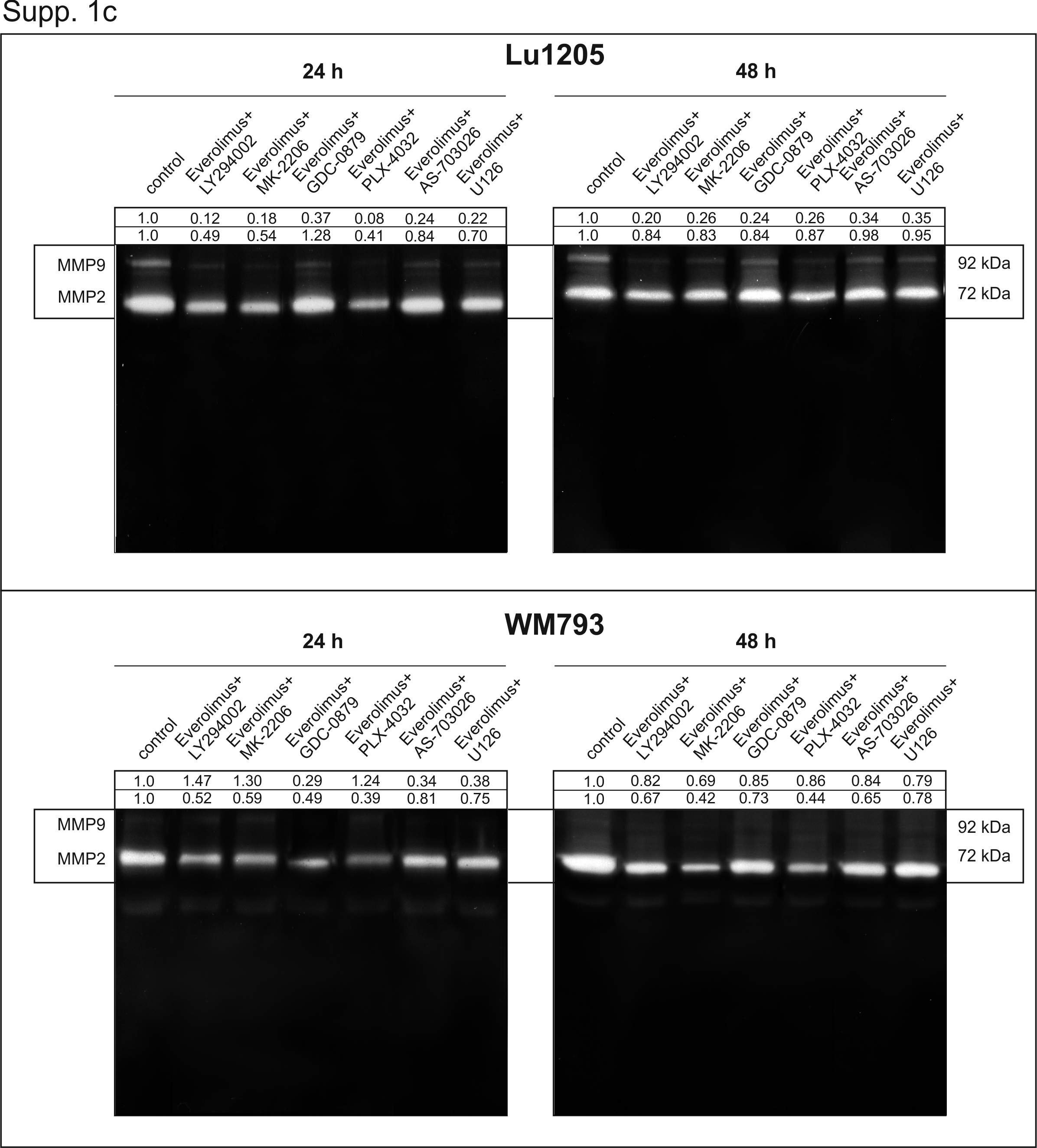

Supplement: Supplementary file 3 — Supplementary material 3 (TIFF 971 kb) [file 13577_2019_270_MOESM3_ESM.tif]

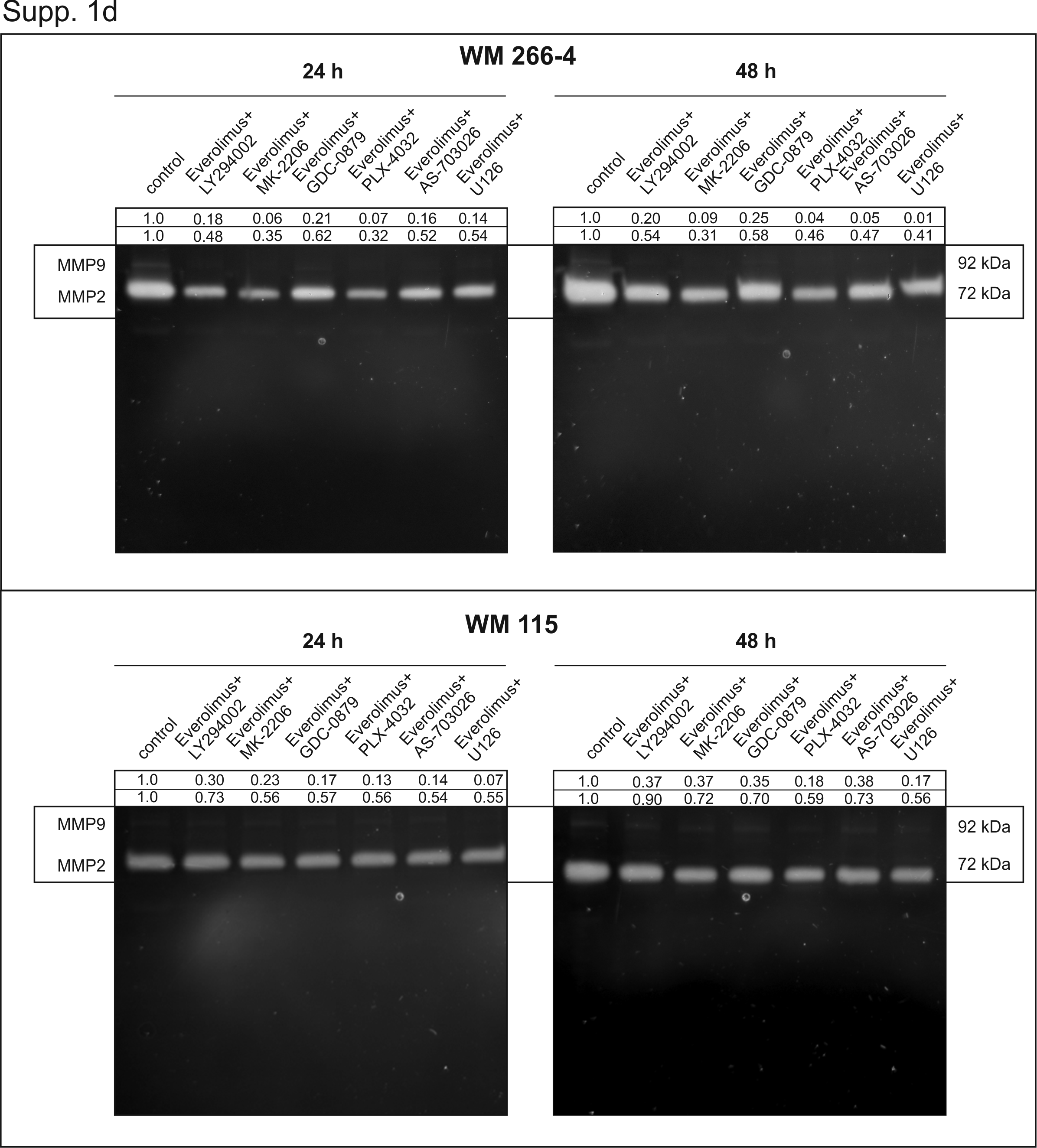

Supplement: Supplementary file 4 — Supplementary material 4 (TIFF 982 kb) [file 13577_2019_270_MOESM4_ESM.tif]
