# Supplementary material for: mTOR inhibitor everolimus reduces invasiveness of melanoma cells
Source: Hum Cell. 2019 Oct 4;33(1):88–97. doi: 10.1007/s13577-019-00270-4 (PMC6965047; doi:10.1007/s13577-019-00270-4)
Supplement: Supplementary file 5 — Supplementary material 5 (DOCX 12 kb) [file 13577_2019_270_MOESM5_ESM.docx]

**Cytotoxicity assay**

Cytotoxicity of: PI3K inhibitor – LY294002 (20 μM), ERK1/2 inhibitor - U0126 (10 μM), mTOR inhibitor – everolimus (5 nM), B-RAF –GDC-0879 (2 μM), MEK – AS-703026 (10 μM), AKT - MK-2206 (2 μM), B-RRAF- PLX-4032 (10 μM) assay was determined using Cytotoxicity Detection Kit LDH, Roche, Germany. In all examined melanoma cell lines inhibitors: LY294002, U0126, Everolimus, GDC-0879, AS-703026, MK-2206, and PLX-4032 showed no cytotoxicity effect tested in a culture medium at the time of 72h. LDH activity in the culture medium in no case exceeded 3.2%.
