# Supplementary material for: mTOR inhibitor everolimus reduces invasiveness of melanoma cells
Source: Hum Cell. 2019 Oct 4;33(1):88–97. doi: 10.1007/s13577-019-00270-4 (PMC6965047; doi:10.1007/s13577-019-00270-4)
Supplement: Supplementary file 6 — Supplementary material 6 (DOCX 12 kb) [file 13577_2019_270_MOESM6_ESM.docx]

**Figure legend:**

**Figure Suppl. 1a-d** The effect of protein kinase inhibitors on gelatinolytic activities of MMP-2 and MMP-9 in melanoma cells.

Densitometric analyses of MMP-2 and MMP-9 activities were performed on raw volume (sum of intensities of bound-volume calculated from the area of the peak). Presented are representative of at least three independent experiments with similar results.
